# In Acute Dengue Infection, High TIM-3 Expression May Contribute to the Impairment of IFNγ Production by Circulating Vδ2 T Cells

**DOI:** 10.3390/v14010130

**Published:** 2022-01-12

**Authors:** Eleonora Cimini, Germana Grassi, Alessia Beccacece, Rita Casetti, Concetta Castilletti, Maria Rosaria Capobianchi, Emanuele Nicastri, Chiara Agrati

**Affiliations:** 1 Laboratory of Cellular Immunology and Pharmacology, National Institute for Infectious Diseases “L. Spallanzani”, Via Portuense 292, 00149 Rome, Italy; eleonora.cimini@inmi.it (E.C.); germana.grassi@inmi.it (G.G.); rita.casetti@inmi.it (R.C.); 2 Clinical Department, National Institute for Infectious Diseases “L. Spallanzani”, Via Portuense 292, 00149 Rome, Italy; alessia.beccacece@inmi.it (A.B.); emanuele.nicastri@inmi.it (E.N.); 3 Laboratory of Virology, National Institute for Infectious Diseases “L. Spallanzani”, Via Portuense 292, 00149 Rome, Italy; concetta.castilletti@inmi.it; 4 Department of Epidemiology, Pre-Clinical Research and Advanced Diagnostic, National Institute for Infectious Diseases “L. Spallanzani”, Via Portuense 292, 00149 Rome, Italy; maria.capobianchi@inmi.it; 5 Saint Camillus International University of Health Sciences, Via di Sant’Alessandro, 8, 00131 Roma, Italy; 6 Department of Infectious Tropical Diseases and Microbiology, IRCCS Sacro Cuore Don Calabria Hospital, Via Don A. Sempreboni 5, 37024 Negrar di Valpolicella, Italy

**Keywords:** dengue infection, innate immune response, Vδ2 T cells, TIM-3, PD-1, exhaustion marker

## Abstract

γδ T cells are innate cells able to quickly eliminate pathogens or infected/tumoral cells by their antiviral and adjuvancy activities. The role of γδ T cells during Dengue Viral Infection (DENV) infection is not fully elucidated. Nevertheless, human primary γδ T cells have been shown to kill in vitro DENV-infected cells, thus highlighting their possible antiviral function. The aim of this work was to characterize the phenotype and function of Vδ2 T cells in DENV patients. Fifteen DENV patients were enrolled for this study and peripheral blood mononuclear cells (PBMC) were used to analyze Vδ2-T-cell frequency, differentiation profile, activation/exhaustion status, and functionality by multiparametric flow cytometry. Our data demonstrated that DENV infection was able to significantly reduce Vδ2-T-cell frequency and to increase their activation (CD38 and HLA-DR) and exhaustion markers (PD-1 and TIM-3). Furthermore, Vδ2 T cells showed a reduced capability to produce IFN-γ after phosphoantigenic stimulation that can be associated to TIM-3 expression. Several studies are needed to depict the possible clinical impact of γδ-T-cell impairment on disease severity and to define the antiviral and immunoregulatory activities of γδ T cells in the first phases of infection.

## 1. Introduction

γδ T cells represent unconventional T lymphocytes that are considered as a bridge between innate and adaptive immunity. Human γδ T cells recognize a wide range of self- and non-self antigens, such as soluble proteins and small peptides, prenyl-pyrophosphates, stress- or infection-induced ligands, and MHC class I chain-related protein A and B (MICA/B) [1]. γδ T cells constitute about 1–10% of total circulating lymphocytes in peripheral blood [2] and the two major γδ-T-cell populations are described, respectively, as Vδ1 and Vδ2 T cells [3]. Vδ1 T cells are abundant in tissues such as intestinal and pulmonary epithelium, in skin, and in the female reproductive tract [4]. Vδ2 T cells make up 90% of circulating γδ T cells in peripheral blood in healthy humans and they are able to quickly interact with pathogens or infected/tumoral cells to eliminate them by cytotoxic activity [5,6,7]. γδ T cells play a role in several clinical settings such as autoimmune diseases [8,9,10], allergies [11,12], and cancer [13], and are known to exert a potent antimicrobial activity against both bacterial [14] and viral infections [15,16]. Moreover, human Vδ2 T cells exert their antimicrobial activities through both cytolytic and non-cytolytic mechanisms [16,17,18,19] and can shape several others immune functions (e.g., dendritic cell activation, neutrophil recruitment/activation, Th1 polarization, soluble factor release, and NK-like cytotoxicity) by producing immune-modulating factors [20].

Among the genus *Flavivirus*, the dengue virus (DENV) belongs to a larger, heterogeneous group of viruses called “Arboviruses”. There are four serotypes, antigenically distinct, of DENV—DEN-1, -2, -3, and -4. Dengue infection severity may depend on several factors and in general results in a flu mild infection. Primary infection with any of the four DENV serotypes could confer long-term protection to the homologous serotype. However, a secondary infection with a different serotype represents one of the major risk factors for severe DENV infection [21]. An impairment of innate immune response has been associated with a more severe DENV infection [22], but the involvement of γδ T cells in protection/pathogenesis is still not well elucidated. In humans, primary γδ T cells can contribute to the immune response during DENV infection by providing an early source of IFN-γ, as well as by killing DENV-infected cells [23], but the modulation of their effector function is not well defined. In this work, we characterized Vδ2 T cells in DENV-infected patients, focusing on their activation and functionality.

## 2. Materials and Methods

### 2.1. Cohort of Patients

Patients returning to Italy from endemic countries were enrolled at the National Institute for Infectious Diseases (INMI), L Spallanzani of Rome, after diagnosis of DENV infection. All patients were enrolled before the COVID-19 pandemic. The local ethical committee approved this study (approval number: 9/2020), and all participants gave written informed consent. Fifteen healthy donors (HD) were enrolled as controls. Clinical data of all patients are reported in Table 1.

### 2.2. Serological and Virological Assays

The DENV diagnosis was performed by using both molecular and serological assays and other arboviral infections (e.g., Zika and Chikungunya viruses) were ruled out. Serum samples were tested by indirect immunofluorescence assay for both IgM and IgG antibodies against ZIKV, DENV, and Chikungunya virus (IFA Arboviral Fever Mosaic 2 IgM and IgG Euroimmun AG, Luebeck, Germany). Molecular assay were performed on serum samples by real-time PCR (RT-PCR) for ZIKV, DENV, and CHIKV RNA [24,25,26]. Virological data are reported in Table 2.

### 2.3. Peripheral Lymphocyte Isolation

Peripheral blood mononuclear cells (PBMC) of both DENV patients and HD were isolated by gradient centrifugation (Lympholyte, Cedarlane), counted in trypan blue and frozen in FBS (fetal bovine serum, Euroclone) with 10% DMSO (Euroclone).

### 2.4. Phenotypic and Intracellular Staining

PBMC from peripheral blood of patients and HD were thawed in complete medium (RPMI-1640 supplemented with 10% fetal bovine serum, 2 mmol glutamine, 50 IU/mL penicillin, and 50 µg/mL streptomycin; EuroClone, Pero, Italy) and suspended 1 × 10^6^ cells/mL. Cell viability was analyzed by trypan blue exclusion. Phenotypic analysis was performed by flow cytometry. Specifically, Vδ2 T-cells were analyzed by using the following anti-human monoclonal antibodies: FITC/PE-conjugated anti-Vδ2, PeC5.5-conjugated anti-CD3, APC-A750-conjugated anti-CD27, APC-conjugated anti-CD38, PE-conjugated anti-perforin, PE-conjugated anti-TIM-3, and APC-conjugated anti-IFN-γ (BD Biosciences, San Jose, CA, USA) and V660-conjugated anti-CD45RA, KrO-conjugated anti-CD45, ECD-conjugated anti-HLA-DR, and Viakrome IR885 (Beckman Coulter, Brea, CA, USA). Briefly, thawed PBMC was incubated with mAbs cocktail for 20 min, washed once with buffer (PBS 1×, 0.1% NaN3, 1% BSA) and fixed with 1× para-formaldehyde (PFA, Sigma, Pero, Italy). Two functional tests were performed by flow cytometry. The cytotoxic potential was tested by analyzing the perforin content by intracellular staining of fixed PBMC. The IFN-γ production by Vδ2 T cells was analyzed after stimulation of PBMC with or without phosphoantigen (PhAg, IPH1101, 3 µM) in the presence of Brefeldin A (10 µg/mL, Serva). Intracellular staining was performed on fixed samples by staining cells with IFN-γ or perforin mAbin buffer (PBS 1×, 0.1% NaN3, 1% BSA, saponin 0.5%) for 20 min at room temperature. After incubation, cells were washed once with buffer (PBS 1×, 0.1% NaN3, 1% BSA, saponin 0.1%). Sample acquisition and data analysis were performed by Cytoflex Flow Cytometer (Beckman Coulter). Data were analyzed with Cytoflex software (BC). Results are shown as box and whiskers—the box encompasses the interquartile range of individual measurements, the horizontal bar-dividing line indicates the median value, and the whiskers represent maximum and minimum values.

### 2.5. Statistical Analysis

Statistical significance of results was determined by Graph Pad Prism software. Statistical analysis was performed using the non-parametric Mann–Whitney assay and differences were considered significant when the *p* value was less than 0.05.

## 3. Results

**Figure 1:**
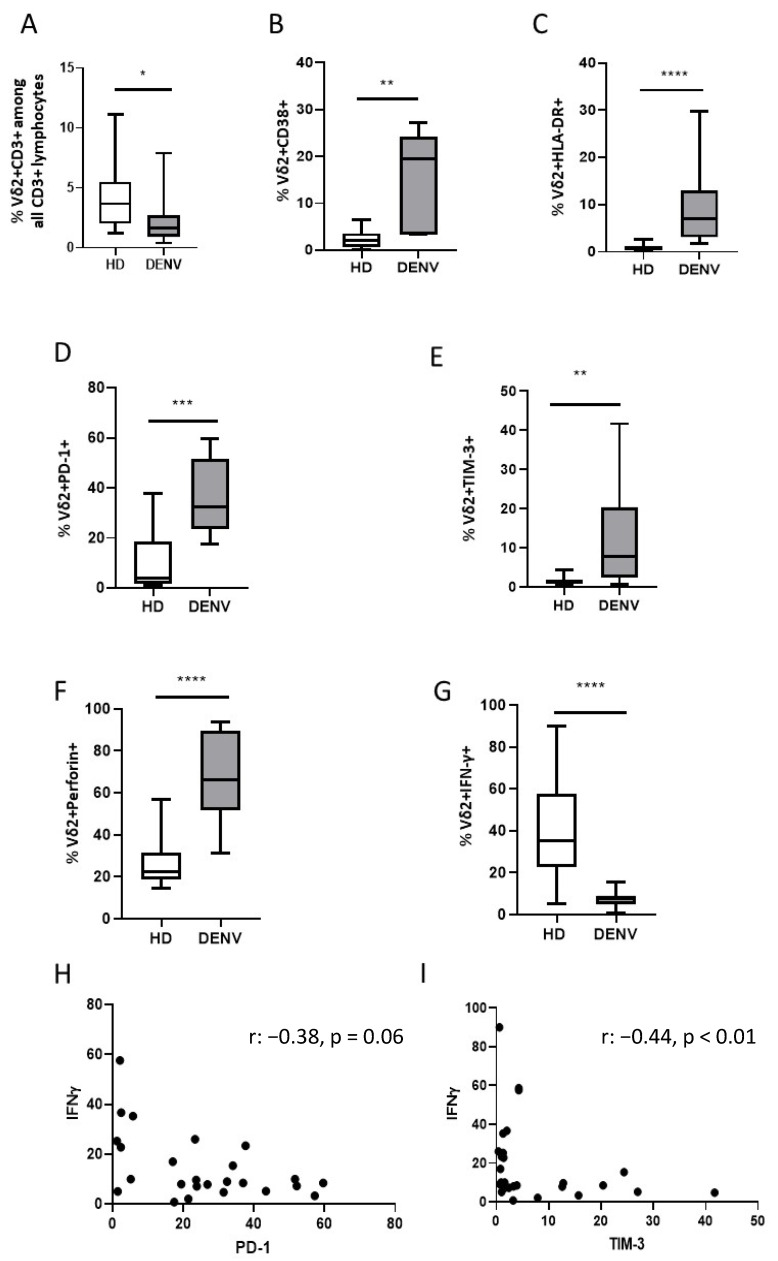
Phenotypic and functionality characteristics of γδ T cells. Frequency (**A**) activation markers (**B**,**C**: CD38, HLA-DR), exhaustion markers (**D**,**E**: PD-1, TIM-3), and functionality (**F**,**G**: perforin, IFN-γ) were performed on PBMC of DENV patients and HD by multiparametric flow cytometry. Correlations (**H**,**I**: IFN-γ/PD-1; IFN-γ/TIM-3) were performed by Spearman test for non-parametric data. *: *p* < 0.05; **: *p* < 0.01; ***: *p* < 0.001; ****: *p* < 0.0001.

To investigate the impact of DENV infection on circulating Vδ2 T cells, we characterized their frequency and differentiation/activation/exhaustion profile and their functionality by multiparametric flow cytometry. A significant reduction of Vδ2-T-cell frequency was observed in DENV patients (DENV vs. HD *p* < 0.05) (Figure 1A). In parallel, a significant Vδ2 T-cell activation was observed, as shown by a significant increase of CD38 (DENV vs. HD, *p* < 0.0001) and of HLA-DR (DENV vs. HD, *p* < 0.0001) expression (Figure 1B,C). In contrast, the differentiation profile of Vδ2 T cells showed no significant differences between patients and HD (data not shown), in accordance with our previous work [27].

We then investigated a possible impact of DENV infection on the expression of exhaustion markers on Vδ2 T cells. Results showed a significantly higher PD-1 (DENV vs. HD, *p* < 0.0002) and TIM-3 (DENV vs. HD, *p* < 0.0029) marker expression in DENV patients compared to HD (Figure 1D,E). The functional analysis revealed a significant enrichment of perforin-positive Vδ2 T cells (DENV vs. HD, *p* < 0.0001) and a reduced frequency of IFN-γ-producing Vδ2 T cells after PhAg stimulation (DENV vs. HD, *p* < 0.0001) when compared to controls. Finally, we evaluated a possible involvement of exhaustion marker expression in reducing the ability to produce IFN-γ after specific stimulation. A significant negative correlation was observed between the frequency of IFN-γ-producing Vδ2 T cells and TIM-3 expression (R: −0.44, *p* < 0.01, Figure 1H) but not with PD-1 marker expression (R: −0.38, *p* = 0.06).

## 4. Discussion

DENV infection is one of the major emerging infectious diseases. Despite a large amount of data on humoral and adaptive T-cell response against DENV, very few data are currently available about the innate immune response. During DENV infection, several innate immune pathways are activated, including type-I interferon, complement, apoptosis, and autophagy [28]. Moreover, the effective functionality of innate cells is associated with milder DENV infection, suggesting a protective role of these innate cells. In DENV-infected patients, an early activation of NK cells was also reported, which was clearly associated with mild DENV diseases [29], suggesting that an early and effective activation of innate cells may be a crucial step in controlling viral replication and disease severity. Indeed, severe infection showed a dysfunctional NK response characterized by a reduction of both IFN-γ and cytotoxic markers [30].

Few data are available about the involvement of γδ T cells in the protection/pathogenesis of DENV infection. γδ T cells killed in vitro DENV-infected cells [23] and contributed to virus clearance in an in vivo mouse model [31], suggesting the ability to mediate a protective immunity. Our results showed that γδ T cells responded to acute DENV infection increasing the expression of activation markers, thus confirming previous published data [30]. Moreover, the γδ-T-cell activation was paralleled by an increase in the expression of exhaustion markers (PD-1 and TIM-3), suggesting a possible induction of a dysfunctional state of these cells. The co-expression of both activation and exhaustion markers in immune cells is well demonstrated in other infections such as Ebola [32,33] and SARS-CoV2 [34], and suggests an over-activation-induced immune impairment. The inhibitory receptor PD-1 exerts a wide range of immunoregulatory roles in T-cell activation and tolerance [35] and represents a marker to define T-cell exhaustion [36]. Accordingly, the PD-1 expression on γδ T cells strongly reduces their effector functions [37]. A similar inhibitory function was also exerted by TIM-3 which is up-regulated both on αβ and γδ T cells after activation [38,39]. Several observations demonstrated a role of both PD-1 and TIM-3 in reducing cytokine production and cytotoxicity of γδ T cells both in viral infection and in cancer [40].

The impact of DENV on γδ-T-cell function has been studied in vitro where primary γδ T cells responded rapidly to autologous DENV-infected dendritic cells by secreting IFN-γ and upregulating CD107a [23]. In contrast, we showed that during acute DENV infection these two functions seemed to be not coordinated, with an effective production of perforin but an impaired IFN-γ production. Indeed, a significant reduction of IFN-γ-producing γδ T cells after PhAg stimulation was shown and was inversely correlated with TIM-3 expression. Accordingly, a role of TIM-3 in γδ-T-cell exhaustion was also shown in malaria patients where the high expression of TIM-3 was linked to a reduced Vδ2-T-cell pro-inflammatory cytokine production [41] and in cancer patients was associated to a reduced killing activity [42]. The direct involvement of TIM-3 in reducing Vδ2-T-cell functionality and its association with the clinical course of the diseases needs further confirmation.

A protective role of γδ T cells was also reported in Zika infection where a massive expansion of effector/activated Vδ2 T-cells occurred [24]. These cells showed, in vitro, a killing capability against ZIKV-infected cells through NKG2D/NKG2DL interaction [5], suggesting a protective role by a TCR-independent mechanism.

Together, these data showed that, on the one hand, DENV infection induced Vδ2 T-cell activation and perforin production but, on the other hand, increased TIM-3 expression, which was associated with a significant reduction of IFN-γ after TCR-mediated stimulation. Several other studies are needed to define a possible association between γδ-T-cell functionality and viral severity.

## Figures and Tables

**Table 1:** Characteristics of 15 DENV patients included in the study.

Age (Mean ± SD) (Years)	Gender (M/F)	Travel History (pts)	Symptom Onset (Range, Days)	Symptoms	DENV RT-PCR	DENV Serotype
42 ± 11	6/9	India (2 pts) Thailand (2 pts) Philippines (2 pts) Singapore (1 pts) Maldives (3 pts) Brazil (1 pts) Indonesia (2 pts) Jamaica (1 pts) Cuba (1 pts)	2–5	Cutaneous rash: 8/15 Fever: 15/15 Headache: 11/15 Arthralgia: 11/15	Positive: 15 Negative: 0	Ser 1: 4 Ser 2: 3 Ser 3: 5 Ser 4: 0 ND: 3

M, male; F, female; pts, patients; DENV, dengue virus; ND, not determined.

**Table 2:** Virological data of 15 DENV patients included in the study.

Patient ID (Gender/DENV Serotype)	Flavivirus PCR	ZIKV RT-PCR	DENV RT-PCR	CHIKV RT-PCR	Anti-ZIKV IgG	Anti-ZIKV IgM	Anti-DENV IgG	Anti-DENV IgM	Anti-CHIKV IgG	Anti-CHIKV IgM
PT1 (F/DENV 1)	Positive	ND	Positive	ND	ND	ND	<1:20	<1:20	ND	ND
PT2 (F/DENV ND)	Positive	ND	Positive	ND	ND	ND	1:20	1:40	ND	ND
PT3 (F/DENV 3)	Positive	Negative	Positive	Negative	<1:20	<1:20	1:80	1:80	<1:20	<1:20
PT4 (F/DENV 3)	Positive	Negative	Positive	Negative	<1:20	<1:20	1:80	1:160	<1:20	<1:20
PT5 (M/DENV 1)	Positive	Negative	Positive	Negative	<1:20	<1:20	1:20	1:80	<1:20	<1:20
PT6 (M/DENV 3)	Positive	Negative	Positive	Negative	<1:20	<1:20	<1:20	1:20	<1:20	<1:20
PT7 (M/DENV ND)	Positive	Negative	Positive	Negative	<1:20	<1:20	1:40	1:80	<1:20	<1:20
PT8 (F/DENV ND)	Positive	Negative	Positive	Negative	<1:20	<1:20	1:160	<1:20	<1:20	<1:20
PT9 (F/DENV 1)	Positive	Negative	Positive	Negative	<1:20	<1:20	<1:20	<1:20	<1:20	<1:20
PT10 (F/DENV 3)	Positive	Negative	Positive	Negative	<1:20	<1:20	<1:80	<1:40	<1:20	<1:20
PT11 (M/DENV 1)	Positive	ND	Positive	Negative	ND	ND	1:320	<1:20	<1:20	<1:20
PT12 (M/DENV 2)	Positive	Negative	Positive	Negative	<1:20	<1:20	<1:20	<1:40	<1:20	<1:20
PT13 (F/DENV 3)	Positive	ND	Positive	Negative	<1:20	<1:20	<1:20	<1:20	<1:20	<1:20
PT14 (M/DENV 2)	Positive	Negative	Positive	Negative	<1:20	<1:20	<1:20	<1:20	<1:20	<1:20
PT15 (F/DENV 2)	Positive	Negative	Positive	Negative	<1:20	<1:20	<1:20	<1:20	<1:20	<1:20

M, male; F, female; DENV, dengue virus; ZIKV, Zika virus; CHIKV, Chikungunya virus; ND, not done. RT-PCR, Real Time PCR.

## Data Availability

The data presented in this study are openly available at http://rawdata.inmi.it, accessed date on 6 January 2022.
